# Pott’s Puffy Tumor in an Adult Female: A Case Report in a Rare Demographic

**DOI:** 10.7759/cureus.24922

**Published:** 2022-05-11

**Authors:** Nathaniel Hitt, Tyler Rosengren, Thomas Delaney, Timothy Dettmer

**Affiliations:** 1 Internal Medicine, MercyOne North Iowa Medical Center, Mason City, USA; 2 Otolaryngology, MercyOne North Iowa Medical Center, Mason City, USA

**Keywords:** pott’s puffy tumor, incidental radiological finding, forehead mass, sinusitis, frontal sinus

## Abstract

Pott’s puffy tumor (PPT) is a localized forehead swelling with underlying subperiosteal abscess formation and osteomyelitis of the frontal bone. It is a rare complication of frontal sinusitis, and it is especially rare in adult females. A careful review of existing literature identified only 17 cases in adult females. Treatment requires antibiotic therapy and often surgical drainage/debridement. Here, we present a case of a 76-year-old female diagnosed with PPT. She was placed on amoxicillin-clavulanate 875 mg twice daily for six weeks. Her symptoms resolved with the antibiotic course, and she is scheduled for otolaryngology (ENT) follow-up, including dedicated sinus computed tomography (CT). While Pott’s puffy tumor is a rare manifestation of chronic sinusitis, it is important to recognize and treat to avoid serious intracranial complications.

## Introduction

Pott’s puffy tumor (PPT) is a localized forehead swelling with underlying subperiosteal abscess formation and osteomyelitis of the frontal bone. It is a rare complication of frontal sinusitis, trauma, or previous surgery [1,2]. It more commonly occurs in children than in adults as a result of underdeveloped frontal sinuses and increased blood flow through the diploic veins. Furthermore, the diploic veins increase the risk of intracranial infection by connecting the frontal sinus mucosa to the dural venous plexus [2]. The male-to-female prevalence ratio has been reported at 3:1 [1]. The increased use of antibiotics has decreased the duration and prevalence of complications of sinusitis, making PPT a rare entity, especially in adult females [1,2]. A search of existing literature revealed only 17 cases in adult females [1,3-7]. We present the case of a 76-year-old female with Pott’s puffy tumor.

## Case presentation

**Figure 1:**
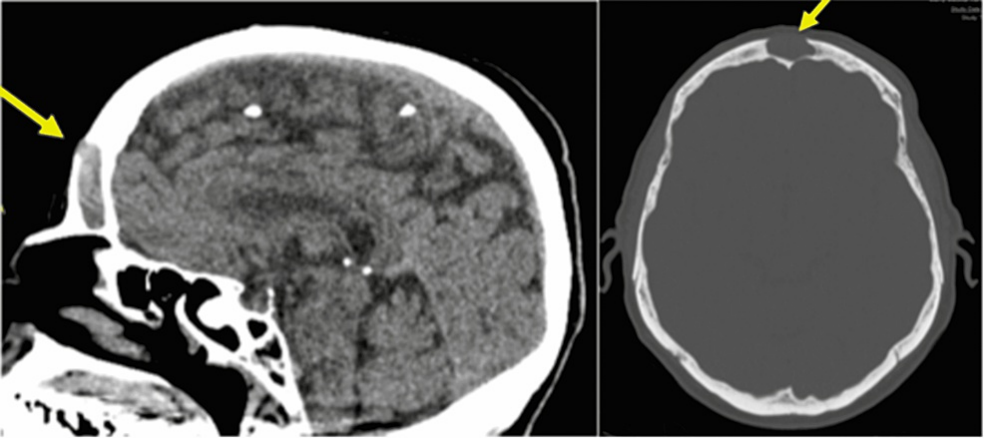
Head CT without contrast There was an expansion of the frontal sinus in the midline with loss of the anterior cortex (arrows). The maxillary sinuses and frontal sinuses were completely opacified with the walls of the maxillary sinuses being sclerotic. The orbits were normal, and the mastoid air cells were clear. There was no intracranial hemorrhage, mass, or CT evidence of infarct.

**Figure 2:**
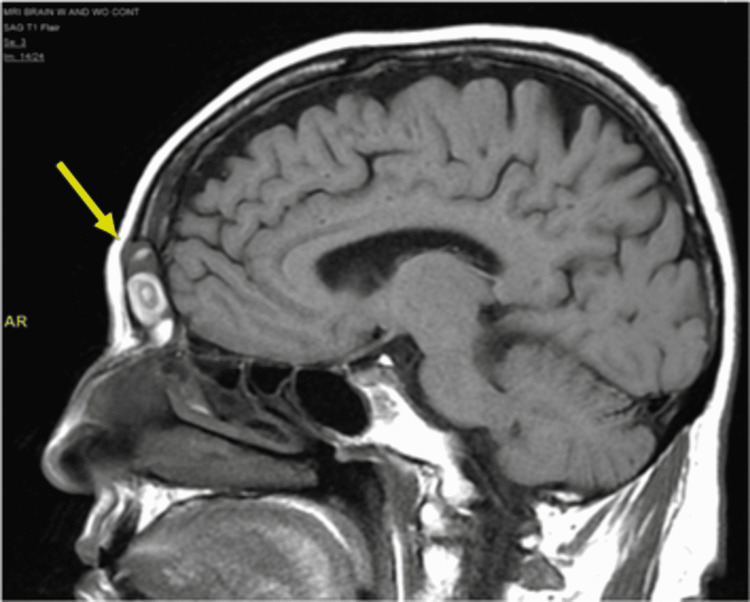
Brain MRI T1/FLAIR Complete opacification of the frontal sinus with disruption of the anterior cortex of the frontal sinus and protuberance of the opacification within the frontal sinus into the subgaleal soft tissues (arrow). No evidence of secondary intracranial involvement.

A 76-year-old female presented to the emergency department with syncope resulting in hospital admission to the medical service. Head computed tomography (CT) performed in the emergency department during the patient’s syncopal workup incidentally revealed opacified maxillary and frontal sinuses and resultant absence of the anterior wall of the frontal sinus consistent with a Pott’s puffy tumor (Figure 1). Follow-up magnetic resonance imaging (MRI) showed complete opacification of the frontal sinuses and probable disruption of the anterior cortex of the frontal sinus with a protuberance of the opacification within the frontal sinus into the subgaleal soft tissues (Figure 2). There was no evidence of secondary intracranial involvement. Further history revealed six months of tender midline forehead swelling. The patient denied overt symptoms of sinusitis. She had no history of sinusitis, sinus surgery, or trauma. Per the patient, she reported having had a previous outpatient incision, and drainage performed by an ophthalmologist expressed caramel-colored fluid without further workup; the date of intervention could not be confirmed. However, the pain and swelling continued. Upon examination, the patient had no obvious midline forehead swelling but was tender to palpation. Examination of the anterior nasal passages was unremarkable.

The otolaryngology service (ENT) evaluated the patient. Nasopharyngeal scope revealed patent nasal passageways without evidence of swelling along the ostiomeatal complexes. Left-sided purulent yellow drainage was noted draining into the nasopharynx from the frontal sinus. Purulent drainage was accumulating along the posterior pharyngeal wall. No polyps or masses were identified. The patient was placed on Augmentin 875 mg twice daily for six weeks. Her swelling and tenderness resolved with the antibiotic course, and she is scheduled for ENT follow-up including a dedicated sinus CT.

## Discussion

Pott’s puffy tumor (PPT) can present at any age but is more common in children ages 6-15 and in males [2,8]. Our patient, a 76-year-old female, represents an uncommon demographic for a rare disease. Symptoms are described as soft tissue forehead swelling accompanied by fever, headache, and rhinorrhea [2,8]. More extensive infections may include symptoms of periorbital swelling, cutaneous drainage, or symptoms of increased intracranial pressure, meningitis, or encephalitis [1,2]. A nasopharyngeal examination can reveal purulent sinus drainage and/or obstruction. Diagnosis requires a high-resolution CT scan to confirm the presence of osteomyelitis, assess the integrity of the posterior wall of the frontal sinus, and evaluate for surgical treatment. MRI is required when an endocranial complication is suspected or must be ruled out [2,8].

The diagnosis in our case was readily apparent as an incidental finding on head CT and brain MRI upon admission. However, in a patient with midline forehead swelling in which PPT is suspected, a thorough history accompanied by a physical examination and nasopharyngeal endoscopy should be performed to confirm the diagnosis and delineate the inciting cause of PPT. A differential diagnosis should include hematoma, more superficial skin and soft tissue infections, and soft tissue tumors and masses [1]. Infection is the most common cause of PPT, followed by trauma. Less common etiologies include surgery, odontogenic infection, cocaine abuse, wrestling injuries, and insect bites. Common etiologies due to pathogens include microbes of both superficial skin infections and sinus infections, including *Streptococcus*, *Staphylococcus*, and anaerobes [2]. Other etiologies include noninfectious masses such as dermoid cysts, hemangiomas, lipomas, epidermal inclusion cysts, and osteomas [9]. The differential should also include intracranial complications, especially with symptoms of increased intracranial pressure, meningitis, or encephalitis. Complications include epidural, subdural, and brain abscess [1,2].

Our patient’s presentation may represent an early diagnosis considering her relatively benign physical examination and symptomatology. She reported typical, but mild, tender midline forehead swelling. However, midline swelling was not readily apparent upon physician examination. Cutaneous swelling may have been reduced by previous outpatient incision and drainage. She had no other symptoms. A complete physical examination revealed no erythema, cutaneous drainage, or neurologic symptoms. The nasopharyngeal examination did reveal purulent drainage and supported a diagnosis of chronic sinusitis. Previous case reports describe presentations similar to our case in which external drainage was performed repeatedly to target what was suspected to be a more superficial soft tissue infection [1]. To avoid misdiagnosis and delay in treatment, otolaryngology (ENT) referral should be considered early in the course of the workup. A recent case series indicated otolaryngologists make the correct diagnosis with a lower incidence of intracranial complications when compared to other specialties [1].

Treatment often requires surgery and antibiotic therapy. In most cases, it is recommended that broad-spectrum intravenous antibiotic therapy be initiated promptly to cover *Streptococcus*, *Staphylococcus*, and anaerobes [2]. If intracranial involvement is present, antibiotics must have blood-brain barrier penetration. Antibiotic therapy can be de-escalated following culture results. Many cases require surgical drainage of the sinus and debridement of any infected bone. Additional surgical management may be required for intracranial involvement such as abscess drainage. Our patient was treated with oral amoxicillin-clavulanate 875 mg twice daily for six weeks. Oral antibiotics were chosen in this case given the patient’s mild symptoms, relatively benign physical examination, lack of involvement of the posterior wall of the frontal sinus, and absence of intracranial complication. Follow-up imaging and surgery were not immediately considered given the patient’s relatively benign presentation and significant comorbidities; the patient’s presenting symptom of syncope was secondary to a high-degree heart block requiring pacemaker placement with the Pott’s puffy tumor being found incidentally during evaluation. Her swelling and tenderness resolved with the antibiotic course, and she is scheduled for otolaryngology follow-up including a dedicated sinus CT and consideration of surgery.

## Conclusions

Pott’s puffy tumor is a rare diagnosis, especially among older females. Given the low number of reported cases in this demographic, treatment strategy and follow-up appear to be based largely upon experience and research with younger patients. Previous literature suggests intravenous antibiotics, but this case shows that oral antibiotics can be appropriate in select cases. While Pott’s puffy tumor is an uncommon manifestation of chronic sinusitis, the severity of the complications warrants early recognition, followed by prompt treatment and follow-up.
